# Cognitive Brain Networks and Enlarged Perivascular Spaces: Implications for Symptom Severity and Support Needs in Children with Autism

**DOI:** 10.3390/jcm14093029

**Published:** 2025-04-27

**Authors:** Stefano Sotgiu, Giuseppe Barisano, Vanna Cavassa, Mariangela Valentina Puci, Maria Alessandra Sotgiu, Angela Nuvoli, Salvatore Masala, Alessandra Carta

**Affiliations:** 1 Division of Child Neuropsychiatry, Department of Medicine, Surgery and Pharmacy, University of Sassari, 07100 Sassari, Italyalessandra.carta@aouss.it (A.C.); 2 Department of Neurosurgery, Stanford University, Stanford, CA 94305, USA; barisano@stanford.edu; 3 Unit of Statistics, Department of Medicine, Surgery and Pharmacy, University of Sassari, 07100 Sassari, Italy; 4 Department of Medicine, Surgery and Pharmacy, University of Sassari, 07100 Sassari, Italy; 5 Radiology Unit, Department of Medicine, Surgery and Pharmacy, University of Sassari, 07100 Sassari, Italy

**Keywords:** autism, brain functional networks, default mode network, central executive network, frontoparietal network, salience network, perivascular spaces, symptoms, needs

## Abstract

**Background/Objectives:** The severity of autism spectrum disorder (ASD) is clinically assessed through a comprehensive evaluation of social communication deficits, restricted interests, repetitive behaviors, and the level of support required (ranging from level 1 to level 3) according to DSM-5 criteria. Along with its varied clinical manifestations, the neuroanatomy of ASD is characterized by heterogeneous abnormalities. Notably, brain MRI of children with ASD often reveals an increased number of perivascular spaces (PVSs) compared to typically developing children. Our recent findings indicate that enlarged PVSs (ePVSs) are more common in younger male patients with severe ASD and that specific ePVS locations are significantly associated with ASD symptoms. **Methods:** In this study, we mapped ePVSs across key regions of three major cognitive networks—the Default Mode Network (DMN), the combined Central Executive/Frontoparietal Network (CEN/FPN), and the Salience Network (SN)—in 36 individuals with different symptom severities and rehabilitation needs due to ASD. We explored how the number, size, and location of PVSs in these networks are related to specific ASD symptoms and the overall need for rehabilitation and support. **Results:** Our results suggest that ePVSs in the DMN, CEN/FPN, and SN are strongly correlated with the severity of certain ASD symptoms, including verbal deficits, stereotypies, and sensory disturbances. We found a mild association between ePVSs and the level of support needed for daily living and quality of life. **Conclusions:** Dysfunction in cognitive networks associated with the presence of ePVSs has a significant impact on the severity of ASD symptoms. However, the need for assistance may also be influenced by other comorbid conditions and dysfunctions in smaller, overlapping brain networks.

## 1. Introduction

Autism spectrum disorder (ASD) is a neurodevelopmental disorder characterized by a range of communication and social reciprocity deficits, along with repetitive behaviors (e.g., stereotypies) and atypical responses to sensory stimuli (e.g., hypo- or hypersensitivity) [1,2]. Early signs of ASD can appear very early in life, although a formal diagnosis is typically made around 18 months of age [3,4]. Diagnosing ASD requires both direct clinical observation [5] and a multidisciplinary approach [6], supported by parental interviews [7,8]. The severity of ASD is categorized by the DSM-5 into three levels based on the amount of support required for daily living [1]. However, the individual need for assistance and intervention often does not take into account the frequent neurodevelopmental comorbidities associated with ASD, such as intellectual disability and attention-deficit hyperactivity disorder (ADHD), as well as other common conditions like anxiety, depression, epilepsy, and sleep disorders [2,9,10].

Alongside the diverse clinical manifestations seen across patients, the neuroanatomy of ASD is characterized by heterogeneous brain abnormalities. These include transient brain enlargement, region-specific volumetric changes on MRI, alterations in the thickness of grey and white matter, and microstructural brain tissue changes observed in neuropathology [11,12,13,14].

Some children with ASD show an excess of extra-axial cerebrospinal fluid (CSF) [15] and reduced diffusivity along the perivascular spaces (PVSs) compared to healthy children [16]. PVSs are pial-lined spaces surrounding the brain’s arterioles and venules, facilitating CSF flow, which is essential for clearing interstitial fluid and waste products from the brain [17]. As the primary pathway of the glymphatic system, PVSs play a crucial role in maintaining brain homeostasis [18]. While PVSs are typically small, they can become visible on MRI when enlarged (ePVSs), due to a reduction in glymphatic flow. This enlargement is often seen in aging, neuroinflammatory, and neurodegenerative conditions [19,20,21]. PVS dilation has also been observed in the brains of children with ASD [22,23,24].

More recently, children who later develop ASD have been found to exhibit an enlargement of PVSs, accompanied by an increased CSF volume during the second year of life [25]. This suggests that PVS enlargement in ASD may indicate impaired CSF flow, potentially leading to stagnation and the accumulation of neurotoxic metabolites, which could contribute to progressive neurodegeneration throughout the brain [12,15,16,17,25,26]. Indeed, individuals with ASD, compared to typically developing children, show atypical patterns of brain connectivity across functional MRI networks [27].

Functionally, these networks are not isolated but interact dynamically, depending on age and the task at hand [28]. Currently, there is no universal taxonomy or brain network atlas for children that fits all circumstances. This is because many networks are large-scale and stable, while others overlap and exhibit varying properties [27,29]. Among the proposed fMRI brain networks (e.g., somatomotor, sensorimotor, language, visual, and limbic networks) [30], those associated with key cognitive functions are likely the most studied, particularly in the context of ASD [31].

The Default Mode Network (DMN) is active during rest and when a person is not engaged in specific tasks. It is thought to play a role in self-referential and internal thoughts. Key regions of the DMN include the medial prefrontal cortex, posterior cingulate cortex, and angular gyrus [32].

The Central Executive Network (CEN) is associated with high-level cognitive functions and works in concert with the overlapping Frontoparietal Network (FPN) [31,33]. While the CEN primarily supports higher-order functions such as working memory, decision making, and problem solving, the FPN is more flexible and adaptive, particularly in shifting between distinct cognitive states. Key regions of the CEN include the dorsolateral prefrontal cortex (DLPFC), posterior parietal cortex, and anterior cingulate cortex. The key regions of the FPN include the DLPFC, posterior parietal cortex, inferior parietal lobule, and prefrontal cortex. As a result, the CEN and FPN share important key regions [33,34]. Additionally, the caudate nucleus, which is connected to the prefrontal cortex, plays a role in regulating the activity of the CEN/FPN. Dysfunction in the caudate nucleus may contribute to difficulties in adapting to new situations or shifting focus [35,36].

Another key cognitive network is the Salience Network (SN), which is essential for emotional regulation. The key regions of the SN include the anterior insula and the anterior cingulate cortex [37].

In summary, several abnormalities in the DMN, CEN/FPN, SN, and other networks have been consistently observed in individuals with ASD [27,38,39,40,41,42]. Under- or over-connectivity within the DMN may be related to difficulties in tasks that require switching between internal and external focus, leading to challenges with self-referential thinking and social cognition. Altered connectivity in the CEN/FPN could contribute to deficits in cognitive flexibility, planning, and decision making [43]. Impaired salience detection within the SN may also contribute to social difficulties, as individuals with autism often struggle with switching between tasks and tend to become overly focused on specific activities [35,37].

In two recent studies, we quantified ePVSs in the brain MRIs of children with ASD and other neuropsychiatric disorders [44] and found that an ePVS is a neuroimaging feature of younger male patients with severe ASD. Additionally, by mapping the number, diameter, and volume of PVSs across 72 brain regions, we found that specific ASD symptoms—such as verbal dysfunction, stereotypies, and abnormal sensory processing—are significantly linked to distinct ePVS localizations. Notably, ePVSs in the two rostral middle frontal regions, part of the DLPFC, were associated with the most severe clinical manifestations of ASD [45].

In this new study, we mapped PVSs in key regions of the cognitive brain networks (DMN, CEN/FPN, and SN) in individuals with ASD and examined how the number, size, and location of the PVSs relate to overall rehabilitation needs and specific ASD symptoms. Symptoms such as language disorders, stereotypies, and abnormal sensory processing were selected due to their association with potentially distinct underlying brain networks.

## 2. Materials and Methods

### 2.1. Study Design and Subjects

This study was approved by the Ethics Committee of Azienda Ospedaliero-Universitaria, Cagliari, Italy (PROT. PG/2023/5144), and conducted in accordance with relevant regulations. Written informed consent was obtained from all parents or legal guardians. Clinical records of consecutive children diagnosed with ASD were retrospectively analyzed at the Child Neuropsychiatry Unit, University Hospital, Sassari, from 2018 to 2022. The diagnosis of ASD, supported by the Autism Diagnostic Observation Schedule, second edition (ADOS-2) [5], has been previously described [44].

All eligible children had previously undergone brain MRI in accordance with guidelines [46], due to abnormal neurological examinations, macrocephaly, dysmorphisms, or EEG abnormalities, as outlined in earlier work [44,45]. Thirty-six children were ultimately included in this study based on the exclusion criteria detailed elsewhere [44,45].

The presence (yes/no) and severity of language impairment, the presence (yes/no) of motor stereotypies, and the presence (yes/no) of sensory distortion were extracted from the specific ADOS-2 subtest scores. The three progressive levels of the patients’ rehabilitation needs were defined according to the DSM-5 guidelines [1].

### 2.2. MRI Processing

Standard MRI acquisition and subsequent PVS mapping were performed as detailed in our previous studies [44,45]. Brain MRI was conducted on all individuals using the same 1.5 Tesla Philips Achieva MRI scanner (Eindhoven, The Netherlands) at the University Hospital in Sassari. The MRI protocol included a T1-weighted spin-echo axial sequence (TR: 357–895 ms; TE: 10–15 ms; flip angle: 69°; resolution: 0.8 × 0.8 × 4.4 mm^3^) and a T2-weighted turbo spin-echo axial sequence (TR: 4000–7000 ms; TE: 100 ms; flip angle: 90°; resolution: 0.4 × 0.4 × 4.4 mm^3^). Both sequences were available for all subjects and used for the segmentation of PVSs.

In brief, white matter (WM) and basal ganglia (BG) masks were generated using a fast, sequence-adaptive whole-brain segmentation algorithm applied to the T1 images [47]. T2 images were then rigidly transformed to the T1 space. Intensity was uniformized across the brain using the 3dUnifize function in AFNI (Analysis of Functional NeuroImages); the program is available free (https://www.opensourceimaging.org/project/afni/) for noncommercial research purposes [48]. We then calculated voxel-wise intensity differences between each voxel and its surrounding voxels in the white matter and basal ganglia. A voxel was defined as a PVS if the intensity difference was greater than 60, as described in our previous study [44,45]. The PVS maps were reviewed blind to the clinical status and demographic information. Because the slice thickness was larger than the PVS thickness, we used the number of voxels rather than the volume in mm^3^ to measure the PVS volumes. Additionally, as PVS volume is highly correlated with WM volume [49], the PVS volume value was normalized by the amount of WM for each individual. PVS voxels were considered part of the same cluster if their edges or corners were in contact.

For qualitative PVS scoring, we used the visual rating scale described by Potter et al. [50], which assigns scores as follows: 0 if PVSs are not visible, 1 for 1–10 PVSs, 2 for 11–20 PVSs, 3 for 21–40 PVSs, and 4 for > 40 PVSs. We then compared our visual PVS scores with the corresponding quantitative measures and found a significant correlation between the two (Spearman R = 0.61, *p* < 0.0001). Quantitative measures offer several advantages over visual scoring, including higher sensitivity, better reproducibility, and the ability to assess more structural properties of PVSs, such as volume and diameter, which are typically not quantifiable via visual rating scales [44,45,51].

### 2.3. Mapping and Measurement of Perivascular Spaces

We designed the study to achieve a detailed mapping of the PVSs, their predominance in specific cognitive networks, and their correlation with reported symptoms and general assistance needs. As a preliminary step, we measured the number, volume, and diameter of the PVSs, along with the volume of the underlying WM in the cortical areas listed below, as well as within the individual basal ganglia regions [44,45,51].

For this purpose, we parcellated brain areas using the Mindboggle-101 dataset, a comprehensive and publicly accessible collection of manually labeled macroscopic anatomy in MRI scans of the human brain [52]. For each hemisphere, we mapped PVSs in the WM underlying the following cortical areas:

- Temporal lobe: entorhinal cortex, parahippocampal gyrus, fusiform gyrus, superior temporal gyrus, middle temporal gyrus, inferior temporal gyrus, transverse temporal gyrus, and temporal pole (8 areas).
- Frontal lobe: superior frontal, middle frontal gyrus (rostral and caudal), inferior frontal gyrus (pars opercularis, pars triangularis, and pars orbitalis), orbitofrontal gyrus (lateral and medial), precentral gyrus, paracentral lobule, and frontal pole (11 areas).
- Parietal lobe: postcentral gyrus, supramarginal gyrus, superior parietal lobule, inferior parietal lobule, and precuneus (5 areas).
- Occipital lobe: lingual gyrus, pericalcarine cortex, cuneus cortex, and lateral occipital cortex (4 areas).
- Cingulate cortex: rostral anterior, caudal anterior, posterior, and isthmus (4 areas).
- BG areas were subdivided, for each side, into the followings: thalamus, caudate, putamen, and pallidus (4 areas).

For each individual, a total of 288 data points were obtained (72 areas, each with 4 variables). Due to the limited sample size, rather than performing a dimensional analysis of specific clinical symptoms, we applied an “extreme-of-outcome” approach to compare clinical and MRI data. Specifically, language impairment, motor stereotypies, and sensory disturbances were analyzed as binary variables (present/absent). For each region, the following three variables were measured: PVS count (number of PVSs), total PVS volume (expressed as the number of PVS voxels), and PVS diameter (in mm).

### 2.4. Brain Functional Networks

**Figure 1:**
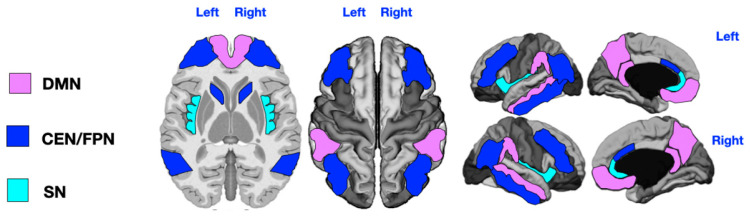
Key regions of the three networks under study. DMN = Default Mode Network (purple: angular gyrus, medial orbitofrontal, middle temporal, posterior cingulate, and precuneus); CEN/FPN = Central Executive and Frontotemporal Networks (dark blue: caudal anterior cingulate, inferior parietal, inferior temporal, lateral orbitofrontal, caudate head, and rostral middle frontal); SN = Salience Network (light blue: insula and rostral anterior cingulate). The DMN key regions are angular gyrus, medial orbitofrontal cortex, middle temporal cortex, posterior cingulate, and precuneus. The CEN/FPN key regions include caudal anterior cingulate, inferior parietal cortex, inferior temporal cortex, lateral orbitofrontal cortex, caudate (head), and rostral middle frontal cortex. The SN key regions are insula and rostral anterior cingulate.

We also analyzed the number, diameter, and volume of PVSs within key cognitive brain networks, as represented in Figure 1, using MRI-based [52] and fMRI-based parcellations [30,53]. Given the shared key regions and substantial functional overlap between the Central Executive Network (CEN) and the Frontoparietal Network (FPN), we arbitrarily merged the key areas of these two networks into one combined network (CEN/FPN), which also included the caudate nucleus [54]. Details of the parcellation are shown in Figure 1.

### 2.5. Statistical Analysis

The completeness and consistency of the collected data, which were stored in an ad hoc database, were evaluated. Sample characteristics were analyzed using descriptive statistics: means and standard deviations (SDs) for normally distributed quantitative variables and medians and interquartile ranges (IQRs) for non-normally distributed quantitative variables. Absolute and relative (percentage) frequencies were used for qualitative variables. The normality of the data was assessed using the Shapiro–Wilk test. In line with assumption’s evaluation, a non-parametric approach was used. After that normality of distribution was assessed through visual inspection and the Shapiro–Wilk test, the comparisons between two groups (with or without symptoms) were conducted using the Mann–Whitney U test. A *p*-value of less than 0.05 was considered statistically significant. All statistical analyses were performed using R software, version 4.4.2.

Additionally, since PVS volume is highly correlated with the volume of the corresponding region [49], we calculated the “PVS volume fraction” and “PVS count fraction” for each region as recommended [50]. These normalized measures were obtained by dividing the PVS volume and count by the number of voxels in the corresponding region, as done in previous studies [44,45]. The collected data were stored in an ad hoc database and are available from the corresponding author upon reasonable request.

## 3. Results

Of the 36 young patients with ASD, 22 (61%) were male, with a mean age of 4 years (SD = 2.3), ranging from 1 to 9 years. Twenty-one children were under the age of 3, and fifteen were over 3 (detailed in Table 1). Seventeen children were not receiving any therapy. Nineteen children were undergoing rehabilitative treatment often in combination: cognitive behavioral therapy (11 children), psychomotor therapy (10), and speech therapy (10). One child was on Methylphenidate for concomitant ADHD, and one was on a combined therapy with Risperidone and Methylphenidate. None of them were on antiepileptic, anti-inflammatory, or immunosuppressive therapy at the time of the MRI.

We were able to confirm the presence of a significant difference in the number and volume of ePVS related to the sex of the individual [44]. This finding is consistent with two of our previous studies in which we have dedicated a particular analysis to comparing ePVS differences between males and females in brain imaging. In the first study [44], we showed, within the same cohort, a significantly higher WM-PVS volume in males compared to females. In the second study, we found that male sex was significantly associated with a higher PVS count and volume also in the BG region [45]. On the contrary, the age of the subjects was not associated with significant differences in PVS enlargement in the cognitive networks, in line with what we reported in a recent study [45].

**Figure 2:**
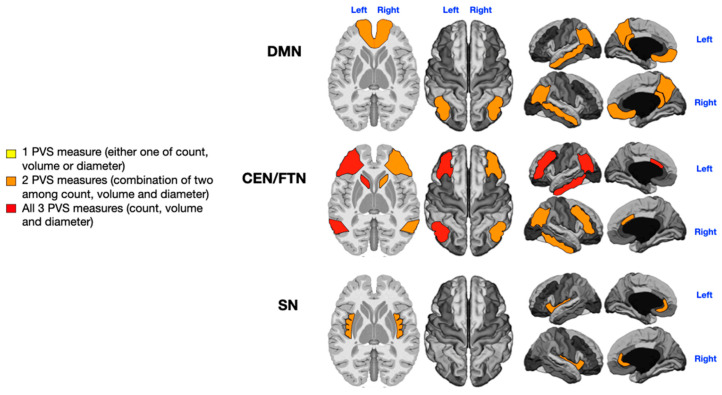
Mapping of PVS enlargement in relation to the degree of language impairment. Colored areas represent significant differences in median values (IQR) of PVS number, volume, and diameter between ASD patients with low vs. high language impairment. Areas are colored based on the extent of PVS abnormalities: yellow = single abnormality (PVS count, volume, or diameter); orange = two PVS abnormalities; red = all three PVS abnormalities. Worse language symptoms are associated with more pronounced PVS alterations, particularly in the left CEN/FPN (red).

**Figure 3:**
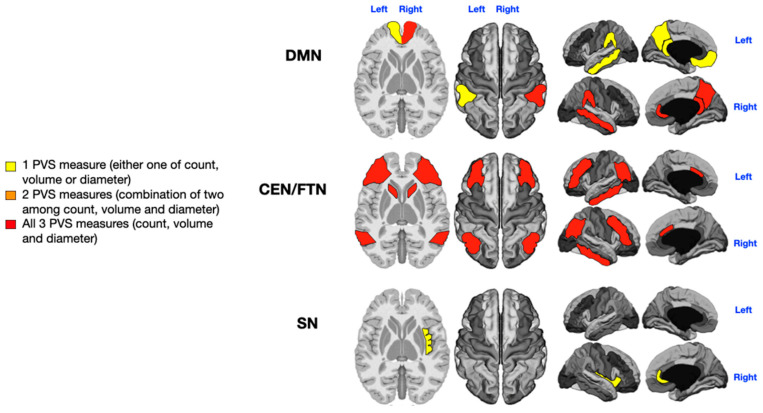
Mapping of PVS enlargement in relation to the presence or absence of sensory disturbances. Colored areas represent significant differences in median values (IQR) of PVS number, volume, and diameter between ASD patients with low vs. high sensory disturbances. Areas are colored based on the extent of PVS abnormalities: yellow = single abnormality (PVS count, volume, or diameter); orange = two PVS abnormalities; red = all three PVS abnormalities. The presence of sensory symptoms is associated with more severe PVS alterations in both the CEN/FPN and the right DMN (red). Minimal enlargement (yellow) in the left DMN and right SN are also significantly associated with sensory issues.

**Figure 4:**
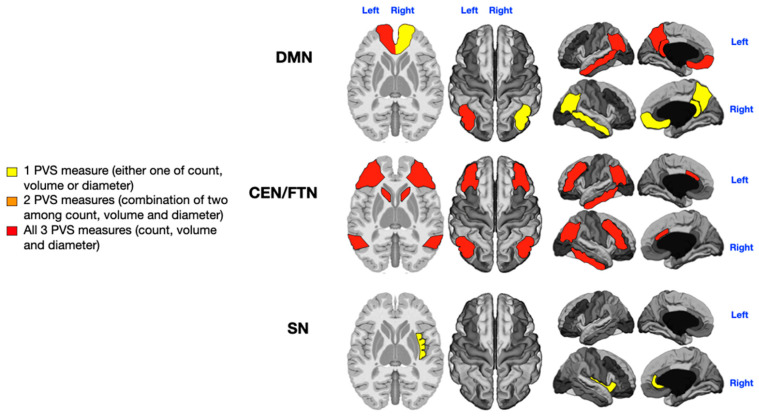
Mapping of PVS enlargement in relation to the presence or absence of motor stereotypies. Colored areas represent significant differences in median values (IQR) of PVS number, volume, and diameter between ASD patients with low vs. high motor stereotypies. Areas are colored based on the extent of PVS abnormalities: yellow = single abnormality (PVS count, volume, or diameter); orange = two PVS abnormalities; red = all three PVS abnormalities. Motor stereotypies are linked to more significant PVS alterations in both the CEN/FPN and the left DMN (red). A minimal enlargement (yellow) in the right DMN and right SN are also significantly associated with motor stereotypies.

**Figure 5:**
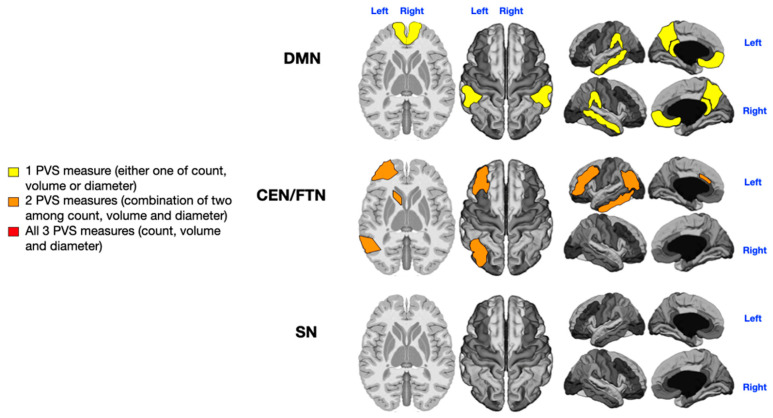
Mapping of PVS enlargement in relation to the level of rehabilitation needs. Colored areas represent significant differences in median values (IQR) of PVS number, volume, and diameter between ASD patients with low vs. high rehabilitation needs. Areas are colored based on the extent of PVS abnormalities: yellow = single abnormality (PVS count, volume, or diameter); orange = two PVS abnormalities; No areas show all three PVS abnormalities (count, volume, and diameter). Higher rehabilitation needs are associated with moderate PVS alterations, especially in the left CEN/FPN (orange). A minimal enlargement (yellow) in both DMNs is also significantly associated with rehabilitation needs.

An analysis of PVSs in the selected cognitive networks (five areas of the DMN, six areas of the CEN/FPN, and two areas of the SN) was performed across the entire patient cohort. The PVS count, diameter, and volume contributed to distinguishing between patients with low and high symptom severity. The findings are depicted in Figure 2, Figure 3, Figure 4 and Figure 5, where colored gradient areas (from yellow to red) represent PVS alterations. Detailed PVS findings for each brain network and statistical results are provided in the Supplementary Materials (Supplementary Table S1 for the DMN, Supplementary Table S2 for the CEN/FPN, and Supplementary Table S3 for the SN).

### 3.1. Language Impairment

Patients were divided into those with no or low impairment (*n* = 17) and those with severe language impairment (i.e., “non-verbal” patients; *n* = 15). The four patients with intermediate language impairment were excluded based on the extreme-of-outcome approach. A significant, direct relationship was found between the severity of language deficits and the number, diameter, and volume of PVSs across all networks. Notably, the left CEN/FPN exhibited significant enlargement of all PVS measures in severely impaired patients (Supplementary Table S2: *p* = 0.005 for PVS count, *p* = 0.01 for PVS diameter, and *p* = 0.029 for PVS volume; Figure 2, red). Additionally, the right and left DMN (*p* = 0.01, *p* = 0.001, *p* = 0.004, and *p* = 0.01; Supplementary Table S1), right CEN/FPN (*p* = 0.028 and *p* = 0.01; Supplementary Table S2), and both SN (*p* = 0.029, *p* = 0.001, *p* = 0.03, and *p* = 0.02; Supplementary Table S3) showed alterations in two PVS measures (Figure 2, orange).

### 3.2. Sensorial Alterations

Patients were categorized as sensorially unimpaired (*n* = 13) or impaired (*n* = 20), irrespective of the type of sensory disturbance (e.g., hearing and smell). Three patients were excluded due to missing data on sensory alterations. A significant, direct relationship was found between sensory impairment and the number, diameter, and volume of PVSs in the CEN/FPN networks (*p* = 0.04, *p* = 0.04, *p* = 0.03, *p* = 0.04, *p* = 0.01, and *p* = 0.003; Supplementary Table S2, red in Figure 3) and in the right DMN (*p* = 0.029, *p* = 0.047, and *p* = 0.046; Supplementary Table S1, red in Figure 3). The left DMN (*p* = 0.01; Supplementary Table S1, yellow in Figure 3) and right SN (*p* = 0.04; Supplementary Table S3) also showed significant but smaller PVS enlargements (yellow in Figure 3).

### 3.3. Motor Stereotypies

Patients were divided into those with (*n* = 17) and without (*n* = 16) motor stereotypies. Three patients were excluded due to missing data on stereotypies. Motor stereotypies were significantly and positively associated with the number, diameter, and volume of PVSs in the left DMN (*p* = 0.01, *p* = 0.048, and *p* = 0.00001; red in Figure 4) and in both the CEN/FPN (*p* = 0.009, *p* = 0.007, *p* = 0.003, *p* = 0.02, *p* = 0.001, and *p* = 0.04; red in Figure 4). The right DMN exhibited significant association with only one PVS measure (*p* = 0.0001; yellow in Figure 4), while the right SN also showed a significant but weak association (*p* = 0.002; Supplementary Table S3, yellow in Figure 4).

### 3.4. Level of Rehabilitation Needs

The 36 patients were stratified according to their rehabilitation needs: low level (ASD level 1; *n* = 10) and high level (ASD level 3; *n* = 20). The remaining six patients, classified as intermediate (ASD level 2), were excluded owing to the extreme-of-outcome strategy, as already described. As shown in Supplementary Tables S1 and S2 and Figure 5, significant relationships were observed between the ASD severity level and PVS number (left DMN) and volume (right DMN) in the WM of both the DMN (*p* = 0.02, *p* = 0.006; Supplementary Table S1, yellow in Figure 5) and the count and diameter of PVSs in the left CEN/FPN (P = 0.014, *p* = 0.018; Supplementary Table S2, orange in Figure 5). No significant PVS enlargement was associated with the ASD severity level in the SN bilaterally (Supplementary Table S3, Figure 5).

## 4. Discussion

The severity of ASD is typically determined through a comprehensive assessment that includes deficits in social communication (both verbal and non-verbal), interaction difficulties, restricted and repetitive behaviors (such as repetitive movements, inflexible behaviors, or distress when routines are disrupted), and the patient’s need for support, as outlined by the DSM-5 classification system (from level 1 to level 3) [1]. However, it is crucial to recognize that ASD is a spectrum, with its severity varying significantly among individuals, even within the same level of need. Comorbid conditions such as ADHD, intellectual disabilities, and epilepsy further complicate the assessment of severity. A reasonable evaluation of ASD severity should be individualized, taking into account clinical assessments, parent/caregiver reports, and a careful estimation of the level of support required for daily functioning [55]. With this background in mind, our findings of altered PVSs in cognitive networks may have important implications for individualized treatment strategies for ASD symptoms.

### 4.1. Language Impairment

Our study demonstrates that the left CEN/FPN is particularly affected by PVS enlargement in children with the most severe language impairments. Moderate PVS enlargements in the bilateral DMN and SN were also associated with language deficits (Figure 2).

The CEN/FPN plays a critical role in language processing, encompassing functions such as production, comprehension, and monitoring speech flow to interpret complex meanings and respond appropriately [56]. These networks collaborate with others, including the DMN and SN, to regulate attention and understanding within linguistic contexts [57]. Dysfunction in the DMN and SN in autism is known to contribute to social communication deficits, especially in interpreting non-verbal and emotional cues in speech [57,58,59,60]. Additionally, the caudate nucleus may exhibit altered connectivity with regions of the CEN, which could further contribute to the language dysfunctions observed in ASD [61].

### 4.2. Sensorial Issues

Sensory processing dysfunction is a hallmark feature of ASD, leading to hypersensitivity, hyposensitivity, unusual sensory-seeking behaviors, or sensory avoidance [62]. In our study, PVS enlargement in the two CEN/FPN networks was significantly associated with the most severe sensory processing problems. Additionally, significant PVS enlargement in the right DMN (indicated by red) and smaller PVS alterations in the right SN (yellow) were observed in patients with more pronounced sensory concerns (Figure 3).

The CEN plays an essential role in processing sensory information and managing sensory inputs, while the SN is crucial for coordinating communication between the DMN and CEN, prioritizing relevant internal and external stimuli [62,63]. This coordination allows the brain to efficiently process sensory events, including emotional states and sensory cues, focusing attention on the most pertinent information. The integration of these functions is essential for adaptive behavior in a dynamic environment [64]. The observed PVS enlargement in these networks may reflect disruptions in the brain’s ability to effectively process sensory information, leading to the sensory difficulties commonly reported in ASD.

### 4.3. Motor Stereotypes

Our findings indicate that the left DMN and both CEN/FPN networks are significantly affected by PVS enlargements in children with motor stereotypies (highlighted in red in Figure 4). Additionally, low-grade (yellow color) PVS enlargement in the right DMN and right SN was also associated with motor stereotypies.

Motor stereotypies in autism are likely driven by altered connectivity between networks that control motor actions, including the basal ganglia, sensory processing networks, and cognitive networks such as the FPN and DMN [65,66]. The basal ganglia, including the caudate nucleus, are essential in regulating voluntary movement and inhibiting unwanted actions. In ASD, dysfunction in striatal connections often leads to excessive or repetitive motor movements [36,67]. The FPN, which is involved in higher-order cognitive processes like cognitive control and task switching, may contribute to difficulties in inhibiting or shifting attention away from repetitive movements. Additionally, dysfunction in the DMN, particularly when these behaviors serve a self-calming function, may further exacerbate motor stereotypies in individuals with autism [60].

### 4.4. Level of Support

In contrast to the significant associations found between PVS enlargement and specific ASD symptoms such as language, sensory, and motor issues, we observed only a low-grade significant association between PVS enlargement and the level of support required by the patients. Specifically, this association was observed as a low-grade PVS enlargement (highlighted in yellow in both DMN regions and orange in the left CEN/FPN), with no notable involvement of the SN (Figure 5).

The DSM-5 [1] defines severity levels based on the need for support, categorizing patients as follows: Level I (requiring support), Level II (requiring substantial support), and Level III (requiring very substantial support). These levels are typically based on core symptoms of ASD. However, it is important to acknowledge that the presentation of these core symptoms is influenced by comorbid conditions, such as intellectual disabilities, anxiety, and depressive disorders, which can significantly impact an individual’s quality of life [55].

The recent literature, including a consensus by Lord et al. [2], introduced the term “profound autism” to describe individuals who are unable to live independently due to co-occurring intellectual disability, language impairment, epilepsy, or self-injury (e.g., hand-biting). Conversely, individuals with minimal or no language or intellectual impairment, without epilepsy or self-injury, can still experience extreme depression, high suicide risk [68], severe anxiety, and hypersensitivity, which may necessitate continuous support from family members or government services.

These factors likely explain why, in our cohort, the estimated severity levels based on required support were only weakly correlated with the dysfunction of cognitive brain networks. The presence of comorbidities and the complexity of individual needs may not be fully captured by a simple level of support measure based on core ASD symptoms alone.

### 4.5. Limitations

Our study has a few important limitations that should be considered when interpreting these findings. First, our study uses a cross-sectional approach to examine MRI patterns of neuroanatomical alterations across patients with varying levels of ASD severity. Additionally, we did not include a control group of age-matched typically developing individuals, which limits our ability to assess the magnitude of PVS dilation in ASD relative to typical development. This also prevents us from determining when PVS formation starts to manifest in ASD and how it compares to neurodevelopment in typically developing children. However, in our first PVS studies [44], we compared children with ASD to non-ASD matched controls in brain PVS imaging. We found that this same cohort of children with ASD showed a higher median PVS volume and count compared to controls. The difference was particularly significant when ASD males were compared to control males. On the other hand, no difference was found in the PVS volume and count between females with ASD and females without ASD [44].

Second, we selected key brain network regions based on an MRI-based parcellation [52] and several fMRI atlases [30,53,69], which are inherently different. The spatial definition of brain networks varies across parcellation methods, meaning that particular regions may be included or excluded depending on the specific approach used. Thus, while our results offer valuable insights, they may differ if other parcellation schemes or atlases were used.

Third, the relatively small sample size, along with the hospital-based case selection, may have introduced a bias towards more severe cases of autism, particularly those requiring MRI due to additional neurological or syndromic symptoms [46]. This could limit the generalizability of the results to the broader ASD population, as milder cases or those without additional comorbidities might not have been represented adequately.

Fourth, we did not perform a multivariate analysis that included covariate correction. Instead, our results are based on bivariate analyses, meaning each variable was analyzed between the two groups without adjusting for covariates. Therefore, factors such as age, sex, medication use, or inflammation-related conditions were not included in a multivariate framework. We acknowledge the relevance of these aspects and will consider them in future research with larger sample sizes and proper multiple comparison adjustments.

Finally, the large amount of data analyzed may raise concerns regarding the casual or causal association between symptoms or assistance needs and the PVS measurements. However, it is important to note that the relationships between increased ePVSs and the severity of symptoms or assistance needs were consistently unidirectional in all our analyses. Specifically, we observed that as ePVSs increased, the severity of symptoms and the level of assistance required also increased, suggesting a clear pattern of association.

## 5. Conclusions

This study examined the neuroanatomical alterations associated with functional connectivity within three large-scale cognitive brain networks: the Default Mode Network (DMN), Salience Network (SN), and Central Executive/Frontal Parietal Network (CEN/FPN). Our findings demonstrate a significant association between enlarged PVSs and the clinical expression of three core symptoms of ASD: language impairment, sensory disturbances, and motor stereotypies. Across the examined networks, the severity of symptomatology was consistently related to the extent of PVS enlargement—measured by count, diameter, and volume—although with some variability in terms of intensity and lateralization. These results align with our previous findings [44,45].

However, we observed only a weak association between PVSs in key regions of the DMN and the left CEN/FPN and the level of support required for independent living. This weaker relationship may reflect that the demands for achieving a higher quality of life in ASD are not solely dependent on the functioning of cognitive networks but also on the dysfunction of smaller-scale overlapping networks, such as sensory, somatomotor, sensorimotor, language, visual, and limbic networks, in addition to comorbid psychiatric and neurological issues [39]. Previous studies have highlighted under-connectivity in regions outside classical cognitive networks, such as the anterior cingulate with eye-controlling areas of the frontal lobe [70], the insula with sensory processing areas [71], and the amygdala with temporal regions [72], which may also contribute to functional impairments in ASD.

In conclusion, our study suggests that ePVSs in the white matter underlying the DMN, CEN/FPN, and SN are strongly linked to the severity of ASD symptoms but only moderately associated with the level of support needed for daily living. These findings underscore the importance of considering cognitive network alterations when developing individualized treatments for ASD symptoms, although further exploration of additional brain networks and comorbid conditions is essential for a comprehensive understanding of the disorder.

## Figures and Tables

**Table 1:** Clinical and demographic information of the cohort of subjects with ASD.

Age (*n* = 36)	Mean = 4 y (SD 2.3) Range = 1–9 y	≤3 y (*n* = 21) >3 y (*n* = 15)	
**Sex (*n* = 36)**	M = 22	F = 14	
**Level of rehab needs** **(*n* = 36)**	Level 1 and 2 (*n* = 16)	Level 3 (*n* = 20)	
**Motor stereotypes (*n* = 33)**	Yes (*n* = 17)	No (*n* = 16)	n.k. (*n* = 3)
**Sensorial disturbances** **(*n* = 33)**	Yes (*n* = 20)	No (*n* = 13)	n.k. (*n* = 3)
**Language impairment** **(*n* = 36)**	Low–moderate (*n* = 21)	Absent language (*n* = 15)	

M = males; F = females; y = years; SD= standard deviation; n.k. = not known.

## Data Availability

The dataset that support the findings of this study are available from the corresponding author, upon reasonable request.

## Supplementary Materials

The following supporting information can be downloaded at https://www.mdpi.com/article/10.3390/jcm14093029/s1, Table S1. Median (IQR) of PVS count, volume and diameter in the right and left DMN, stratified by language disorders, sensorial overload, motor stereotypies, and rehabilitation needs. In bold significant *p* values; Table S2. Median (IQR) of PVS count, volume and diameter in the right and left CEN/FPN, stratified by language disorders, sensorial overload, motor stereotypies, and rehabilitation needs. In bold significant *p* values; Table S3. Median (IQR) of PVS count, volume and diameter in the right and left SN, stratified by language disorders, sensorial overload, motor stereotypies, and rehabilitation needs. In bold significant *p* values.

## Abbreviations

ADHD	Attention deficit hyperactivity disorder
ADOS-2	Autism Diagnostic Observation Schedule, second edition
ASD	Autism spectrum disorder
CEN	Central Executive Network
CSF	Cerebrospinal fluid
DLPFC	Dorsolateral prefrontal cortex
DMN	Default Mode Network
DSM-5	Diagnostic and Statistical Manual of Mental Disorders, Fifth Edition
ePVS	Enlarged perivascular space
fMRI	Functional magnetic resonance imaging
FPN	Frontoparietal Network
MRI	Magnetic resonance imaging
PVS	Perivascular space
SN	Salience Network
WM	White matter

## References

[1] American Psychiatric Association aAPAD-TF (2013). Diagnostic and Statistical Manual of Mental Disorders: DSM-5.

[2] Lord C., Charman T., Havdahl A., Carbone P., Anagnostou E., Boyd B., Carr T., de Vries P.J., Dissanayake C., Divan G. (2022). The Lancet Commission on the future of care and clinical research in autism. Lancet.

[3] Zuckerman K.E., Broder-Fingert S., Sheldrick R.C. (2021). To reduce the average age of autism diagnosis, screen preschoolers in primary care. Autism.

[4] Hadders-Algra M. (2022). Emerging signs of autism spectrum disorder in infancy: Putative neural substrate. Dev. Med. Child Neurol..

[5] McCrimmon A., Rostad K. (2013). Test Review: Autism Diagnostic Observation Schedule, Second Edition (ADOS-2) Manual (Part II): Toddler Module. J. Psychoeduc. Assess..

[6] dos Santos C.L., Barreto I.I., Floriano I., Tristão L.S., Silvinato A., Bernardo W.M. (2024). Screening and diagnostic tools for autism spectrum disorder: Systematic review and meta-analysis. Clinics.

[7] Lord C., Rutter M., Le Couteur A. (1994). Autism Diagnostic Interview-Revised: A revised version of a diagnostic interview for caregivers of individuals with possible pervasive developmental disorders. J. Autism Dev. Disord..

[8] Schopler E., Reichler R.J., DeVellis R.F., Daly K. (1980). Childhood Autism Rating Scale (CARS, CPRS). APA PsycTests.

[9] Carta A., Fucà E., Guerrera S., Napoli E., Valeri G., Vicari S. (2020). Characterization of Clinical Manifestations in the Co-occurring Phenotype of Attention Deficit/Hyperactivity Disorder and Autism Spectrum Disorder. Front. Psychol..

[10] Lai M.C., Kassee C., Besney R., Bonato S., Hull L., Mandy W., Szatmari P.P., Ameis S.H. (2019). Prevalence of co-occurring mental health diagnoses in the autism population: A systematic review and meta-analysis. Lancet Psychiatry.

[11] Brambilla P., Hardan A., di Nemi S.U., Perez J., Soares J.C., Barale F. (2003). Brain anatomy and development in autism: Review of structural MRI studies. Brain Res. Bull..

[12] Nordahl C.W., Lange N., Li D.D., Barnett L.A., Lee A., Buonocore M.H., Simon T.J., Rogers S., Ozonoff S., Amaral D.G. (2011). Brain enlargement is associated with regression in preschool-age boys with autism spectrum disorders. Proc. Natl. Acad. Sci. USA.

[13] Donovan A.P.A., Basson M.A. (2016). The neuroanatomy of autism—A developmental perspective. Am. J. Anat..

[14] Lamanna J., Meldolesi J. (2024). Autism Spectrum Disorder: Brain Areas Involved, Neurobiological Mechanisms, Diagnoses and Therapies. Int. J. Mol. Sci..

[15] Shen M.D., Nordahl C.W., Li D.D., Lee A., Angkustsiri K., Emerson R.W., Rogers S.J., Ozonoff S., Amaral D.G. (2018). Extra-axial cerebrospinal fluid in high-risk and normal-risk children with autism aged 2–4 years: A case-control study. Lancet Psychiatry.

[16] Li X.B., Ruan C.M., Zibrila A.I., Musa M., Wu Y.M., Zhang Z.M., Liu H., Salimeen M. (2022). Children with autism spectrum disorder present glymphatic system dysfunction evidenced by diffusion tensor imaging along the perivascular space. Medicine.

[17] Wardlaw J.M., Benveniste H., Nedergaard M., Zlokovic B.V., Mestre H., Lee H., Doubal F.N., Brown R., Ramirez J., MacIntosh B.J. (2020). Perivascular spaces in the brain: Anatomy, physiology and pathology. Nat. Rev. Neurol..

[18] Yu L., Hu X., Li H., Zhao Y. (2022). Perivascular Spaces, Glymphatic System and MR. Front. Neurol..

[19] Kamagata K., Saito Y., Andica C., Uchida W., Takabayashi K., Yoshida S., Hagiwara A., Fujita S., Nakaya M., Akashi T. (2022). Noninvasive Magnetic Resonance Imaging Measures of Glymphatic System Activity. J. Magn. Reson. Imaging.

[20] Duering M., Biessels G.J., Brodtmann A., Chen C., Cordonnier C., de Leeuw F.-E., Debette S., Frayne R., Jouvent E., Rost N.S. (2023). Neuroimaging standards for research into small vessel disease—Advances since 2013. Lancet Neurol..

[21] Granberg T., Moridi T., Brand J.S., Neumann S., Hlavica M., Piehl F., Ineichen B.V. (2020). Enlarged perivascular spaces in multiple sclerosis on magnetic resonance imaging: A systematic review and meta-analysis. J. Neurol..

[22] Taber K.H., Shaw J.B., Loveland K.A., Pearson D.A., Lane D.M., Hayman L.A. (2004). Accentuated Virchow-Robin spaces in the centrum semiovale in children with autistic disorder. J. Comput. Assist. Tomogr..

[23] Zeegers M., Van Der Grond J., Durston S., Nievelstein R.J., Witkamp T., Van Daalen E., Buitelaar J., Van Engeland H. (2006). Radiological findings in autistic and developmentally delayed children. Brain Dev..

[24] Boddaert N., Zilbovicius M., Philipe A., Robel L., Bourgeois M., Barthélemy C., Seidenwurm D., Meresse I., Laurier L., Desguerre I. (2009). MRI findings in 77 children with non-syndromic autistic disorder. PLoS ONE.

[25] Garic D., McKinstry R.C., Rutsohn J., Slomowitz R., Wolff J., MacIntyre L.C., Weisenfeld L.A.H., Kim S.H., Pandey J., John T.S. (2023). Enlarged Perivascular Spaces in Infancy and Autism Diagnosis, Cerebrospinal Fluid Volume, and Later Sleep Problems. JAMA Netw. Open.

[26] Hayden M.R. (2024). Brain endothelial cell activation and dysfunction associate with and contribute to the development of enlarged perivascular spaces and cerebral small vessel disease. Histol. Histopathol..

[27] Uddin L.Q., Castellanos F.X., Menon V. (2024). Resting state functional brain connectivity in child and adolescent psychiatry: Where are we now?. Neuropsychopharmacology.

[28] Supekar K., Musen M., Menon V. (2009). Development of large-scale functional brain networks in children. PLoS Biol..

[29] Uddin L.Q., Betzel R.F., Cohen J.R., Damoiseaux J.S., De Brigard F., Eickhoff S.B., Fornito A., Gratton C., Gordon E.M., Laird A.R. (2023). Controversies and progress on standardization of large-scale brain network nomenclature. Netw. Neurosci..

[30] Dadi K., Varoquaux G., Machlouzarides-Shalit A., Gorgolewski K.J., Wassermann D., Thirion B., Mensch A. (2020). Fine-grain atlases of functional modes for fMRI analysis. NeuroImage.

[31] Blume J., Dhanasekara C.S., Kahathuduwa C.N., Mastergeorge A.M. (2024). Central Executive and Default Mode Networks: An Appraisal of Executive Function and Social Skill Brain-Behavior Correlates in Youth with Autism Spectrum Disorder. J. Autism Dev. Disord..

[32] Menon V. (2023). 20 years of the default mode network: A review and synthesis. Neuron.

[33] Zhang Y., Lin L., Zhou D., Song Y., Stein A., Zhou S., Xu H., Zhao W., Cong F., Sun J. (2024). Age-related unstable transient states and imbalanced activation proportion of brain networks in people with autism spectrum disorder: A resting-state fMRI study using coactivation pattern analyses. Netw. Neurosci..

[34] Zhu J.-S., Gong Q., Zhao M.-T., Jiao Y. (2025). Atypical brain network topology of the triple network and cortico-subcortical network in autism spectrum disorder. Neuroscience.

[35] Miller E.K., Cohen J.D. (2001). An integrative theory of prefrontal cortex function. Annu. Rev. Neurosci..

[36] Turner K.C., Frost L., Linsenbardt D., McIlroy J.R., Müller R.-A. (2006). Atypically diffuse functional connectivity between caudate nuclei and cerebral cortex in autism. Behav. Brain Funct..

[37] Uddin L.Q. (2015). Salience processing and insular cortical function and dysfunction. Nat. Rev. Neurosci..

[38] Lin P., Zang S., Bai Y., Wang H. (2022). Reconfiguration of Brain Network Dynamics in Autism Spectrum Disorder Based on Hidden Markov Model. Front. Hum. Neurosci..

[39] Hong S.-J., Vogelstein J.T., Gozzi A., Bernhardt B.C., Yeo B.T., Milham M.P., Di Martino A. (2020). Toward neurosubtypes in autism. Biol. Psychiatry.

[40] Di Martino A., Yan C.-G., Li Q., Denio E., Castellanos F.X., Alaerts K., Anderson J.S., Assaf M., Bookheimer S.Y., Dapretto M. (2013). The autism brain imaging data exchange: Towards a large-scale evaluation of the intrinsic brain architecture in autism. Mol. Psychiatry.

[41] Kennedy D.P., Courchesne E. (2008). The intrinsic functional organization of the brain is altered in autism. NeuroImage.

[42] Guo X., Zhai G., Liu J., Zhang X., Zhang T., Cui D., Zhou R., Gao L. (2023). Heterogeneity of dynamic synergetic configurations of salience network in children with autism spectrum disorder. Autism Res..

[43] Park S., Thomson P., Kiar G., Castellanos F.X., Milham M.P., Bernhardt B., Di Martino A. (2024). Delineating a Pathway for the Discovery of Functional Connectome Biomarkers of Autism. Adv. Neurobiol..

[44] Sotgiu M.A., Jacono A.L., Barisano G., Saderi L., Cavassa V., Montella A., Crivelli P., Carta A., Sotgiu S. (2023). Brain perivascular spaces and autism: Clinical and pathogenic implications from an innovative volumetric MRI study. Front. Neurosci..

[45] Sotgiu S., Cavassa V., Puci M.V., Sotgiu M.A., Turilli D., Jacono A.L., Nuvoli A., Masala S., Barisano G., Carta A. (2025). Enlarged perivascular spaces under the dorso-lateral prefrontal cortex and severity of autism. Sci. Rep..

[46] NICE—National Institute for Health and Clinical Excellence Autism—Quality Standard (QS51) 2014 (n.d.). https://www.nice.org.uk/guidance/qs51.

[47] Puonti O., Iglesias J.E., Van Leemput K. (2016). Fast and sequence-adaptive whole-brain segmentation using parametric Bayesian modeling. NeuroImage.

[48] Cox R.W. (1996). AFNI: Software for analysis and visualization of functional magnetic resonance neuroimages. Comput. Biomed. Res..

[49] Barisano G., Sheikh-Bahaei N., Law M., Toga A.W., Sepehrband F. (2021). Body mass index, time of day and genetics affect perivascular spaces in the white matter. J. Cereb. Blood Flow Metab..

[50] Potter G.M., Chappell F.M., Morris Z., Wardlaw J.M. (2015). Cerebral perivascular spaces visible on magnetic resonance imaging: Development of a qualitative rating scale and its observer reliability. Cerebrovasc. Dis..

[51] Barisano G., Lynch K.M., Sibilia F., Lan H., Shih N.-C., Sepehrband F., Choupan J. (2022). Imaging perivascular space structure and function using brain MRI. NeuroImage.

[52] Klein A., Tourville J. (2012). 101 labeled brain images and a consistent human cortical labeling protocol. Front. Neurosci..

[53] Schaefer A., Kong R., Gordon E.M., Laumann T.O., Zuo X.-N., Holmes A.J., Eickhoff S.B., Yeo B.T.T. (2018). Local-global parcellation of the human cerebral cortex from intrinsic functional connectivity MRI. Cereb. Cortex.

[54] Greene D.J., Marek S., Gordon E.M., Siegel J.S., Gratton C., Laumann T.O., Gilmore A.W., Berg J.J., Nguyen A.L., Dierker D. (2020). Integrative and Network-Specific Connectivity of the Basal Ganglia and Thalamus Defined in Individuals. Neuron.

[55] Waizbard-Bartov E., Fein D., Lord C., Amaral D.G. (2023). Autism severity and its relationship to disability. Autism Res..

[56] Ye Z., Zhou X. (2009). Executive control in language processing. Neurosci. Biobehav. Rev..

[57] Sherman L.E., Rudie J.D., Pfeifer J.H., Masten C.L., McNealy K., Dapretto M. (2014). Development of the default mode and central executive networks across early adolescence: A longitudinal study. Dev. Cogn. Neurosci..

[58] Adolphs R. (2001). The neurobiology of social cognition. Curr. Opin. Neurobiol..

[59] Gordon E.M., Laumann T.O., Marek S., Raut R.V., Gratton C., Newbold D.J., Greene D.J., Coalson R.S., Snyder A.Z., Schlaggar B.L. (2020). Default-mode network streams for coupling to language and control systems. Proc. Natl. Acad. Sci. USA.

[60] Maximo J.O., Cadena E.J., Kana R.K. (2014). The Implications of Brain Connectivity in the Neuropsychology of Autism. Neuropsychol. Rev..

[61] Crinion J., Turner R., Grogan A., Hanakawa T., Noppeney U., Devlin J.T., Aso T., Urayama S., Fukuyama H., Stockton K. (2006). Language control in the bilingual brain. Science.

[62] Baron-Cohen S., Ashwin E., Ashwin C., Tavassoli T., Chakrabarti B. (2009). Talent in autism: Hyper-systemizing, hyper-attention to detail and sensory hypersensitivity. Philos. Trans. R. Soc. B Biol. Sci..

[63] Koziol L.F., Budding D.E., Chidekel D. (2011). Sensory integration, sensory processing, and sensory modulation disorders: Putative functional neuroanatomic underpinnings. Cerebellum.

[64] Menon V., Uddin L.Q. (2010). Saliency, switching, attention and control: A network model of insula function. Brain Struct. Funct..

[65] Wang Y., Xu L., Fang H., Wang F., Gao T., Zhu Q., Jiao G., Ke X. (2023). Social Brain Network of Children with Autism Spectrum Disorder: Characterization of Functional Connectivity and Potential Association with Stereotyped Behavior. Brain Sci..

[66] LeMonda B.C., Holtzer R., Goldman S. (2012). Relationship between executive functions and motor stereotypies in children with Autistic Disorder. Res. Autism Spectr. Disord..

[67] Di Martino A., Kelly C., Grzadzinski R., Zuo X.-N., Mennes M., Mairena M.A., Lord C., Castellanos F.X., Milham M.P. (2011). Aberrant striatal functional connectivity in children with autism. Biol. Psychiatry.

[68] Kõlves K., Fitzgerald C., Nordentoft M., Wood S.J., Erlangsen A. (2021). Assessment of suicidal behaviors among individuals with autism spectrum disorder in Denmark. JAMA Netw. Open.

[69] Eickhoff S.B., Yeo B.T.T., Genon S. (2018). Imaging-based parcellations of the human brain. Nat. Rev. Neurosci..

[70] Agam Y., Joseph R.M., Barton J.J., Manoach D.S. (2010). Reduced cognitive control of response inhibition by the anterior cingulate cortex in autism spectrum disorders. NeuroImage.

[71] Ebisch S.J., Gallese V., Willems R.M., Mantini D., Groen W.B., Romani G.L., Buitelaar J.K., Bekkering H. (2010). Altered intrinsic functional connectivity of anterior and posterior insula regions in high-functioning participants with autism spectrum disorder. Hum. Brain Mapp..

[72] Monk C.S., Weng S.-J., Wiggins J.L., Kurapati N., Louro H.M., Carrasco M., Maslowsky J., Risi S., Lord C. (2010). Neural circuitry of emotional face processing in autism spectrum disorders. J. Psychiatry Neurosci..

