# Supplementary material for: Cognitive Brain Networks and Enlarged Perivascular Spaces: Implications for Symptom Severity and Support Needs in Children with Autism
Source: J Clin Med. 2025 Apr 27;14(9):3029. doi: 10.3390/jcm14093029 (PMC12072625; doi:10.3390/jcm14093029)
Supplement: Supplementary file 1 [file jcm-14-03029-s001.zip › jcm-3526182-supplementary.pdf]

Supplementary Table 1. Median (IQR) of PVS count, volume and diameter in the right and left DMN, stratified by language disorders, sensorial overload, motor stereotypies, and rehabilitation needs. In bold significant p values.

**Language impairment**	**Left DMN**	**Right DMN**	**p-value left**	**p-value right**
**No (n =17)**				
PVS count	1 (0-4)	1 (0-4)		
PVS diameter	1.24 (0-1.7)	1.24 (0-1.56)		
PVS volume	1 (0-8)	0 (0-12)		
**Yes (n= 15)**				
PVS count	2 (0.75-8)	3 (0.75-9)	**0.012**	**0.0014**
PVS diameter	1.42 (0-1.6)	1.42 (1.24-1.68)	0.26	**0.031**
PVS volume	8 (0-12)	3 (0-9)	**0.004**	0.17
**Sensory disorder**				
**No (n =13)**				
PVS count	1 (0-4,75)	1 (0-6,75)		
PVS diameter	1.24 (0-1.68)	1.24 (0-1.56)		
PVS volume	1 (0-8)	0 (0-5)		
**Yes (n= 20)**				
PVS count	2 (0-6)	2 (0.5-7)	**0.011**	**0.029**
PVS diameter	1.45 (0-1.63)	1.46 (0-1.68)	0.076	**0.047**
PVS volume	2 (0-11)	3 (0-11)	0.12	**0.046**
**Stereotypes**				
**No (n= 16)**				
PVS count	1 (0-4)	1 (0-6)		
PVS diameter	1.24 (0-1.56)	1.24 (0-1.59)		
PVS volume	0 (0-2)	1 (0-10)		
**Yes (n= 17)**				
PVS count	2 (0-6)	2 (0-6.25)	**0.015**	0.26
PVS diameter	1.52 (0-1.7)	1.46 (0-1.68)	**0.048**	0.15
PVS volume	3.5 (0-12)	7 (1-26)	**0.000018**	**0.00018**
**Level of needs**				
**Level 1 (n= 10)**				
PVS count	1 (0-3)	1 (0-6)		

PVS diameter	1.24 (0-1.58)	1.24 (0-1.55)		
PVS volume	1 (0-13)	0 (0-15)		
**Level 3 (n= 20)**				
PVS count	2 (0-6)	2 (0-6)	**0.02**	0.49
PVS diameter	1.5 (0-1.71)	1.43 (0-1.68)	0.09	0.15
PVS volume	3 (0-11)	5.5 (1-15.25)	0.22	**0.006**

PVS= Perivascular spaces; IQR= interquartile range; n= number of patients; DMN= Default mode network. Unit of measures: PVS count (number); PSV volume (expressed as number of PVS voxels); PSV diameter (expressed as mm).

Supplementary Table 2. Median (IQR) of PVS count, volume and diameter in the right and left CEN/FPN, stratified by language disorders, sensorial overload, motor stereotypies, and rehabilitation needs. In bold significant p values.

Language impairment	Left CEN/FTN	Right CEN/FTN	p-value left	p-value right
**No (n =17)**				
PVS count	0 (0-1)	0 (0-1)		
PVS diameter	0 (0-1.4)	0 (0-1.24)		
PVS volume	0 (0-5)	1 (0-7)		
**Yes (n= 15)**				
PVS count	1 (0-2)	1 (0-3)	**0.028**	**0.005**
PVS diameter	0 (0-1.52)	1.24 (0-1.56)	0.33	**0.013**
PVS volume	2 (0-14)	2 (0-17)	**0.014**	**0.029**
**Sensory disorder**				
**No (n =13)**				
PVS count	0 (0-1)	0 (0-1)		
PVS diameter	0 (0-1.42)	0 (0-1.24)		
PVS volume	0 (0-5)	1 (0-7)		
**Yes (n= 20)**				
PVS count	0 (0-2)	1 (0-2)	**0.044**	**0.043**
PVS diameter	1.24 (0-1.49)	1.24 (0-1.66)	**0.034**	**0.041**
PVS volume	2 (0-15.5)	4 (0-16)	**0.013**	**0.0031**
**Stereotypes**				
**No (n= 16)**				
PVS count	0 (0-1)	0 (0-1)		
PVS diameter	0 (0-1.24)	0 (0-1.31)		
PVS volume	0 (0-5)	1 (0-7)		
**Yes (n= 17)**				
PVS count	1 (0-2)	1 (0-2)	**0.009**	**0.0074**
PVS diameter	0.62 (0-1.56)	1.24 (0-1.53)	**0.0039**	**0.022**
PVS volume	2.5 (0-18.25)	2 (0-14.75)	**0.0017**	**0.042**
**Level of needs**				
**Level 1 (n= 10)**				
PVS count	0 (0-4)	1 (0-2)		

PVS diameter	0 (0-0.93)	1.24 (0-1.32)		
PVS volume	0 (0-6.5)	1 (0-9.5)		
**Level 3 (n= 20)**				
PVS count	1 (0-4)	1 (0-2)	**0.014**	0.25
PVS diameter	0 (0-1.55)	1.24 (0-1.55)	**0.018**	0.24
PVS volume	2 (0-16.5)	2 (0-15.5)	0.056	0.16

PVS= Perivascular spaces; IQR= interquartile range; n= number of patients; CEN/FTN= Central Executive and Frontoparietal networks. Unit of measures: PVS count (number); PSV volume (expressed as number of PVS voxels); PSV diameter (expressed as mm).

Supplementary Table 3. Median (IQR) of PVS count, volume and diameter in the right and left SN, stratified by language disorders, sensorial overload, motor stereotypies, and rehabilitation needs. In bold significant p values.

Language impairment	Left SN	Right SN	p-value left	p-value right
**No (n =17)**				
PVS count	0 (0-3)	1 (0-4)		
PVS diameter	0.62 (0-1.43)	1.24 (0-1.4)		
PVS volume	1 (0-4)	1 (0-8.75)		
**Yes (n= 15)**				
PVS count	1 (0.25-4.75)	5 (2-9.75)	**0.029**	**0.0014**
PVS diameter	1.40 (1.24-1.56)	1.47 (1.4-1.56)	**0.032**	**0.021**
PVS volume	3 (0-6)	4 (0-9.5)	0.21	0.28
**Sensory disorder**				
**No (n =13)**				
PVS count	1 (0-2.25)	1 (0-5)		
PVS diameter	1.24 (0-1.42)	1.24 (0-1.44)		
PVS volume	1 (0-4.25)	8.5 (4-11)		
**Yes (n= 20)**				
PVS count	1 (0-4)	2 (0-4)	0.13	0.15
PVS diameter	1.37 (0-1.51)	1.47 (1.24-1.56)	0.17	**0.042**
PVS volume	2.5 (0-6)	8 (5-16.75)	0.25	0.43
**Stereotypes**				
**No (n= 16)**				
PVS count	1 (0-3)	1 (0-3)		
PVS diameter	1.24 (0-1.46)	1.24 (0-1.46)		
PVS volume	1 (0-4)	1 (0-6.75)		
**Yes (n= 17)**				
PVS count	1 (0-3.25)	2 (0-5)	0.24	0.14
PVS diameter	1.34 (0-1.5)	1.42 (0-1.56)	0.26	0.15
PVS volume	3 (0-8.5)	9 (2.75-12.25)	0.062	**0.0028**
**Level of needs**				
**Level 1 (n= 10)**				
PVS count	1 (0-3)	1 (0-3,25)		

PVS diameter	1.24 (0-1.47)	1.24 (0-1.44)		
PVS volume	1 (0-6.5)	2 (0-9.75)		
**Level 3 (n= 20)**				
PVS count	1 (0-4)	2 (0-5)	0.17	0.16
PVS diameter	1.35 (0-1.5)	1.41 (0-1.56)	0.25	0.12
PVS volume	3 (0-6.25)	4.5 (0-11.25)	0.42	0.45

PVS= Perivascular spaces; IQR= interquartile range; n= number of patients; SN= Salience network. Unit of measures: PVS count (number); PSV volume (expressed as number of PVS voxels); PSV diameter (expressed as mm).
